# ATP1A1 Integrates AKT and ERK Signaling via Potential Interaction With Src to Promote Growth and Survival in Glioma Stem Cells

**DOI:** 10.3389/fonc.2019.00320

**Published:** 2019-04-30

**Authors:** Yang Yu, Chen Chen, Gang Huo, Jinmu Deng, Hongxin Zhao, Rui Xu, Li Jiang, Song Chen, Shali Wang

**Affiliations:** ^1^Institute of Neuroscience, Basic Medical College, Chongqing Medical University, Chongqing, China; ^2^Department of Neurosurgery, The First Affiliated Hospital of Chongqing Medical University, Chongqing, China; ^3^Department of Neurosurgery, Children's Hospital of Chongqing Medical University, Chongqing, China; ^4^Department of Neurosurgery, First Affiliated Hospital, Zunyi Medical College, Zunyi, China

**Keywords:** ATP1A1, GSCs, proliferation, survival, ERK/AKT, Src

## Abstract

Glioma stem cells (GSCs) have been considered to be responsible for treatment failure due to their self-renewal and limitless proliferative property. Recently, the Na^+^/K^+^-ATPase a1 (ATP1A1) subunit was described as a novel therapeutic target for gliomas. Interestingly, our previous proteomics study revealed that ATP1A1 is remarkably overexpressed in GSCs. In the current study, we investigated the role of ATP1A1 in regulating growth, survival, and tumorigenicity of primary human GSCs and the underlying molecular mechanism. We tested RNA and protein expression of ATP1A1 in glioma tissues and GSCs. In addition, we knocked down ATP1A1 in GSCs and assessed the effects thereof on growth, survival, and apoptosis. The role of ATP1A1 in signaling pathways was investigated *in vitro*. We found that the ATP1A1 expression level was associated with the grade of glioma. Knockdown of ATP1A1 in GSCs *in vitro* inhibited cell proliferation and survival, increased apoptosis, and halted cell-cycle progression at the G1 phase. Cell proliferation and survival were resumed upon rescue of ATP1A1 expression in ATP1A1-knockdown GSCs. The ERK1/2 and AKT pathways were inhibited through suppression of Src phosphorylation by ATP1A1 knockdown. Collectively, our findings suggest that ATP1A1 overexpression promotes GSC growth and proliferation by affecting Src phosphorylation to activate the ERK1/2 and AKT signaling pathways.

## Introduction

Glioblastoma (GBM) is the most devastating and lethal primary brain cancer (1, 2). Despite advances in aggressive surgery, radio-, and chemotherapy, GBM therapies are still limited and only palliative (3). The cancer stem cell (CSC) theory, which states that CSCs are a minor population of tumor cells that are multipotent and have self-renewal capacity, and contribute to tumor regrowth and therapy resistance, opens up new approaches to cancer therapy (4). Increasing evidence shows that GSCs may play an integral role in initiating tumor development and facilitating malignant progression (5, 6). Therefore, GSCs are a promising target for studying the molecular pathogenesis of GBM and for clinical treatment. To identify novel targets uniquely expressed in GSCs, we previously performed a proteomics study, which revealed that ATP1A1 (Na^+^/K^+^ ATPase α1 subunit) is abnormally overexpressed in U251GSCs compared to differentiated cells (7), indicating that ATP1A1 may be associated with the intrinsic characteristics and oncogenic phenotype of GSCs. Accordingly, in this study, we firstly studied the role of ATP1A1 in the function and molecular mechanism in GSCs.

The Na^+^/K^+^ ATPase sodium pump is widely distributed in mammalian cell membranes and contributes to many important physiological processes, including maintenance of the ion balance and cellular osmotic pressure (8). Na^+^/K^+^-ATPase is an integral membrane protein. There are four isoforms of the α-subunit of the sodium pump, i.e., α1, α2, α3, α4, which are encoded by *ATP1A1, ATP1A2, ATP1A3*, and *ATP1A4*, respectively (9). The α1 isoform is the predominant isoform in humans. Recent studies have revealed that the sodium pump is closely associated with cancer progression, and the α1 subunit may play a crucial role in the underlying signaling pathway. Inhibition of ATP1A1 reportedly induces apoptosis and impairs migration in GBM cells (10–12). In addition, the sodium pump was found to affect differentiation in bone marrow stromal cell and mouse embryonic stem cells (13, 14). However, the roles of ATP1A1 in GSCs and its underlying molecular mechanisms have not clarified.

The Src family of non-receptor tyrosine kinases (SFKs) is involved in cancer progression in various cancers. SFKs exist in various isoforms, including Src, Fyn, Yes, and Lyn. Among them, Src is the most important member. It functions in activation of the MAPK and PI3K signaling pathways and regulates cell proliferation and growth in various cancers (15–17). Amongst Src-mediated signaling pathways, the interaction between ATP1A1 and Src may be a potential target for the treatment of GBM and other Src-related diseases (18).

In this study, to improve our understanding of the role of ATP1A1 in the malignant phenotype and pathogenesis of GSCs, we evaluated ATP1A1 expression in GBMs of different grades and in two primary GSC lines established from human GBM tissues. We evaluated the role of ATP1A1 in GSC growth, its interactions with Src, and the activation of the ERK1/2 and AKT pathways. Our results revealed that ATP1A1 acts as an oncogene in our GSC models and targeting ATP1A1/Src may suppress GSC proliferation and growth.

## Materials and Methods

### Cell Isolation and Culture

Human brain GBM tissues were from pathologically confirmed surgical specimens and normal human brain tissue was resected from non-neoplastic brain tissue due to hemorrhage, and them were collected at the Department of Neurosurgery of The First Affiliated Hospital of Chongqing Medical University. Primary cell cultures of GBM were established from the fresh GBM tissues. Briefly, tumors were immediately dissected and enzymatically dissociated into single cells, and red blood cells were lysed using PBS/water (1:3, v/v). GSCs were obtained through culturing the cells in serum-free medium (SFM) supplemented with DMEM/F12, 20 ng/ml basic fibroblast growth factor (bFGF, PeproTech, Rocky Hill, NJ, USA), 20 ng/ml epidermal growth factor (EGF, PeproTech, Rocky Hill, NJ, USA), B27 supplement (0.5 × ; Invitrogen, Carlsbad, CA, USA), and 10 ng/ml leukemia inhibitory factor (LIF). Differentiated GBM cells were obtained by culturing the GSCs in Dulbecco's modified Eagle's medium (DMEM) supplemented with 10% fetal bovine serum, 100 mg/ml penicillin, and 100 mg/ml streptomycin in a 5% CO_2_ incubator for at least 7 days. For neurosphere culture of GSCs, uncoated plastic dishes were used. For adherent culture of GSCs, the plates were precoated with laminin (Invitrogen, 5 μg/ml) and polylysine (Sigma, 5 μg/ml), as previously described (19).

### RNA Extraction and Real-Time Quantitative PCR

Total RNA was extracted from cells using TRIzol (TakaRa, Dalian, China), following the manufacturer's instructions. The RNA was reverse-transcribed into cDNA using a PrimeScript^TM^ RT reagent kit (TaKaRa). qPCR was conducted using a SYBR Green RT-PCR kit (TaKaRa). All reactions were run in triplicate and experiments were repeated at least twice. Relative mRNA levels were calculated using the 2^−ΔΔ*CT*^ method and LightCycler 480 SW1.5 software (Roche). The primers for *ATP1A1* were 5′- GGCAGTGTTTCAGGCTAACCAG−3′ (forward) and 5′- TCTCCTTCACGGAACCACAGCA−3′ (reverse); and primers for *GAPDH* were 5′- TGACTTCAACAGCGACACCCA−3′ (forward) and 5′- CACCCTGTTGCTGTAGCCAAA−3′ (reverse).

### Western Blotting

Detailed procedures and the antibodies used are described in the Supplementary Information. Briefly, cells and tissue samples were lysed in lysis buffer and the proteins were quantified. Equal amounts of protein were electrophoresed and transferred to membranes. After blocking of the membranes, they were incubated with primary and secondary antibodies. Protein bands were visualized. GAPDH was used as a normalization control.

### Immunocytochemistry

Cells were plated on glass coverslips precoated with laminin and polylysine, fixed, permeabilized, blocked, and stained with antibodies as described in the Supplementary Information.

### Lentiviral Vector Production and Cell Infection

Short hairpin RNAs (shRNAs) of human ATP1A1 and Src in lentivirus gene transfer vector encoding green fluorescent protein (GFP) were purchased from Origene (Rockville, MD, USA). The most effective shRNA sequences were selected (sh-ATP1A1 and sh-SRC). Lentivirus-GFP without shRNA served as a negative control (sh-NC). Cells (5 × 10^6^) were transfected using a 2-ml mixture composed of 1 × 10^8^ to 1 × 10^9^ viral particles, 8 μg/ml polybrene, and enhanced infection solution. Twenty-four hours after infection, the medium was replaced with fresh medium. To obtain steady knockdown cells, infected cells were selected in medium containing puromycin (6 μg/ml) for 4–7 days and then were propagated in medium containing puromycin (3 μg/ml).

### Transfection of Cells With the Full-Length *ATP1A1* Gene and Inhibitor Treatment

We used the Ras inhibitor farnesylthiosalicylic acid (FTS) at 12.5 μmol/l, and the Src inhibitor 4-amino-5-(4-chlorophenyl)-7-(tbutyl) pyrazolo[3,4-d]pyrimidine (PP2) at 20 μmol/l. sh-ATP1A1 GSCs were transfected with the pCMV6-ATP1A1 or pCMV6-control vector (Origene) using Lipofectamine 3000 (Invitrogen, Carlsbad, CA, USA), according to the manufacturer's protocol. Briefly, cells were seeded in six-well plates. When cultured to 80–90% confluence, the cells were transfected with 2 μg of pCMV6-ATP1A1 or pCMV6-control vector. After 48 h of incubation, the medium was replaced with medium containing FTS or PP2. After culture for an additional 48 h, the cells were harvested for assays. To obtain stable transfectants, after 48 h of incubation, the transfected cells were selected in SFM containing G418 (Sigma-Aldrich, St. Louis, MO, USA; 400 μg/ml for GBM GSCs1 and 800 μg/ml for GBM GSCs2) for 2 weeks.

### BrdU Incorporation and CCK-8 Assays

Cell proliferation and viability were assayed by BrdU incorporation and CCK-8 assays, respectively, as described in the Supplementary Information.

### Flow-Cytometric Analysis of Cell Cycle and Apoptosis

Cell cycle and apoptosis were analyzed by flow cytometry. Details are provided in the Supplementary Information.

### GSC Tumorigenicity Assays in Athymic Nude Mice

Six to eight-week-old, female, athymic nude mice were obtained from the Chongqing Medical University and were housed in a specific pathogen-free environment at Chongqing Medical University. GSCs were injected subcutaneously into the flank of athymic nude mice (2 × 10^6^ cells/mouse and *n* = 5 mice/group) and imaged weekly.

### Immunohistochemical Tissue Microarray Analysis

Tissue microarrays containing cancerous and matched normal tissues were purchased from US Biomax (Rockville, MD USA). Tissue samples were provided as microarrays (catalog Nos. GL722 and GL807). Immunohistochemistry procedures are described in the Supplementary Information. ATP1A1 expression was quantified by counting the glioma cells that positively reacted with anti-ATP1A1.

### Coimmunoprecipitation

Cells were lysed in a buffer containing 1% Nonidet P40, 0.25% sodium deoxycholate, 1 mM EDTA, 150 mM NaCl, 1 mM phenylmethylsulfonyl fluoride, 1 mM sodium orthovanadate, 1 mM NaF, 10 μg/ml aprotinin, 10 μg/ml leupeptin, and 50 mM Tris-HCl (pH 7.4). Cell debris was removed by centrifugation at 20,000 × g at 4°C for 18 min, and the supernatants were immunoprecipitated with anti-ATP1A1, anti-SRC, or anti-IgG antibody. The complexes were pulled down with protein G agarose and analyzed by western blotting.

### Statistical Analysis

All statistical analyses were conducted using GraphPad Prism software (La Jolla, CA, USA). Means of two groups were compared using Student's *t*-test, for multiple comparisons, we used ANOVA. *P* < 0.05 was considered statistically significant. Error bars in figures represent the standard deviation (SD).

## Results

### ATP1A1 Expression Is Upregulated in GBM and GSCs

**Figure 1:**
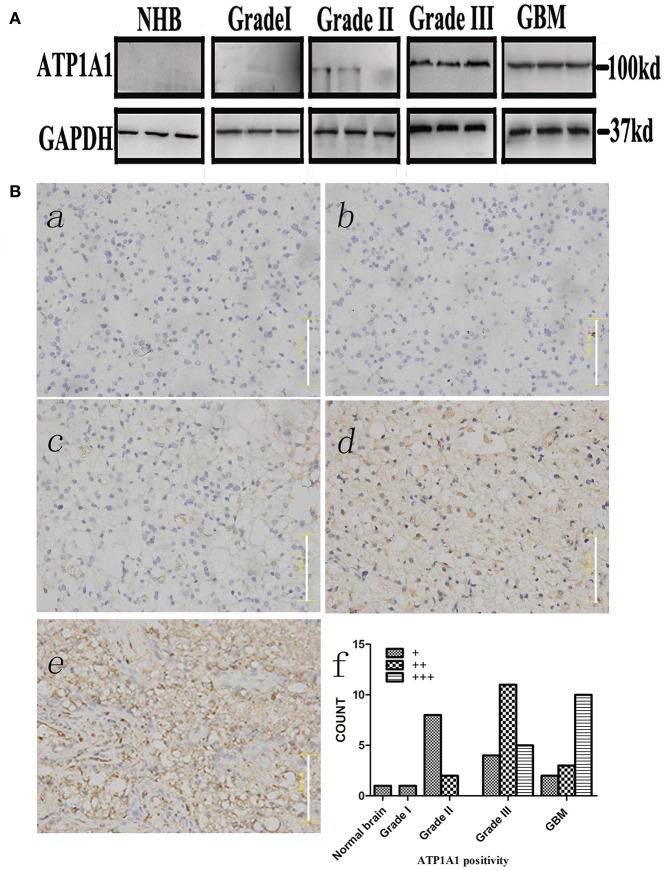
ATP1A1 is overexpressed in high-grade glioma tissues. **(A)** ATP1A1 expression was analyzed by immunoblotting in three GBM samples, three WHO grade III astrocytomas, three WHO grade II gliomas, three WHO grade I gliomas, and normal human brain tissue. **(B)** ATP1A1 expression was analyzed by immunohistochemical staining in gliomas of different grades in tissue arrays. ATP1A1 was slightly stained in normal brain tissue (a), WHO grade I astrocytomas (b), and WHO grade II astrocytomas (c). In contrast, moderate ATP1A1 staining was observed in WHO grade III astrocytomas (d) and GBM WHO grade IV samples (e). Hematoxylin counterstaining was used to visualize nuclei. Scale bar, 100 μm. (f) Bars represent the numbers of tumor samples that fall into each staining category for each tumor grade.

**Figure 2:**
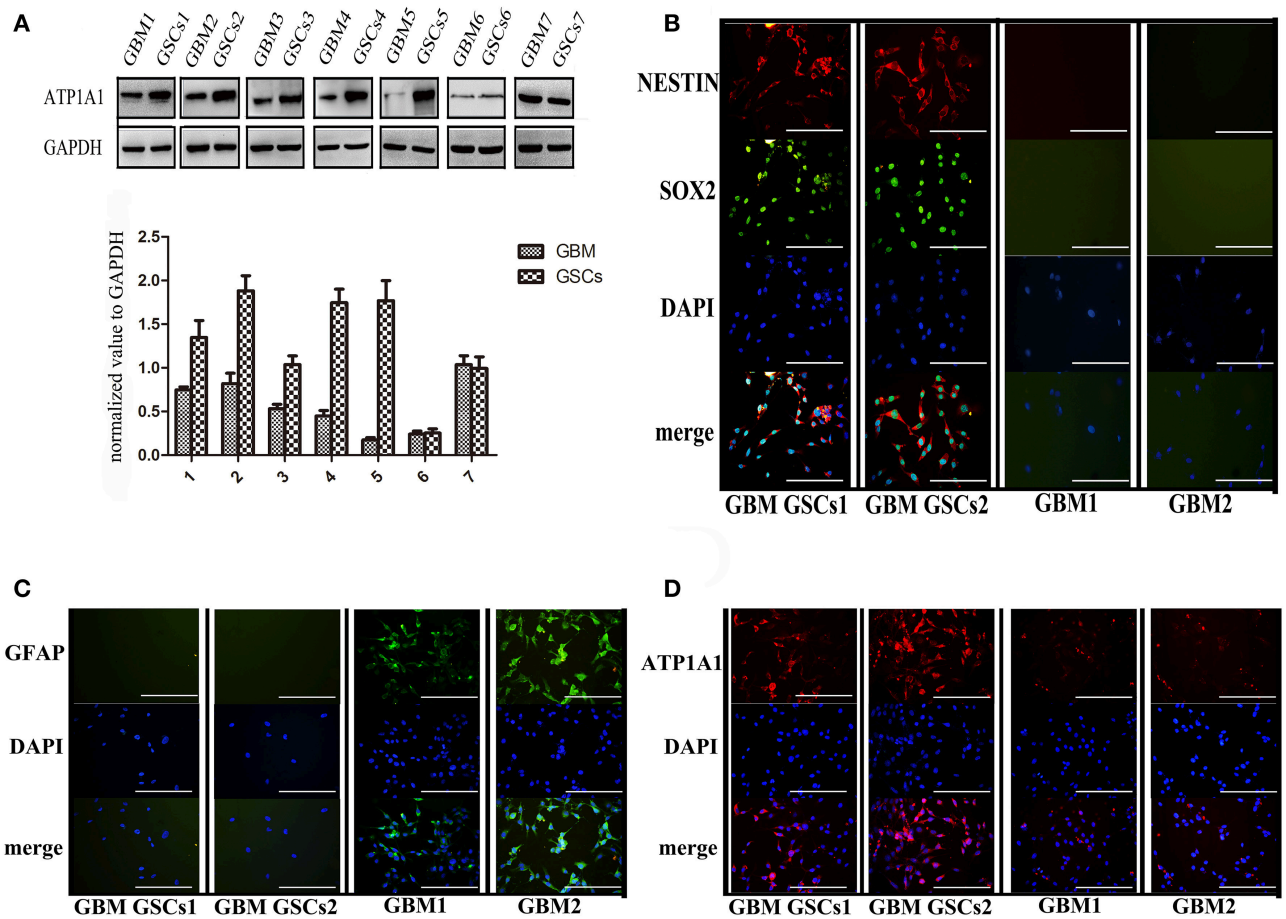
ATP1A1 is overexpressed in primary GSCs, which express stemness markers nestin and SOX2. **(A)** ATP1A1 protein expression was assessed by immunoblotting for seven pairs of patient-matched GSCs and differentiated glioma cells. **(B)** Representative images of immunocytochemical staining for nestin (red) and SOX2 (green) in GSCs, for GFAP (green) in differentiated GBM cells **(C)**, and for ATP1A1 (red) in GSCs and differentiated GBM cells **(D)**. Nuclei were counterstained with 4′,6′-diamidino-2-phenylindole (blue). Scale bars, 200 μm. Error bars represent the SD, ^*^P < 0.05, ^**^P < 0.01 **(B)**.

To validate our previous findings that ATP1A1 is abnormally overexpressed in U251GSCs compared to differentiated cells (7), we firstly determined ATP1A1 expression levels in GBM tissues of different grades. Both western blotting (Figure 1A) and immunohistochemical data suggested that ATP1A1 was significantly upregulated in high-grade gliomas (WHO grade III astrocytomas and GBMs) when compared with low-grade gliomas (grade I and, II astrocytomas) and normal human brain. Next, we immunohistochemically stained paraffin-embedded gliomas, including 15 GBM tissue arrays (Figure 1Be), 20 grade III astrocytoma tissue arrays (Figure 1Bd), 10 grade II astrocytoma tissue arrays (Figure 1Bc), **five** samples of grade I astrocytoma (Figure 1Bb), and **eight** samples of normal brain (Figure 1Ba). The percentage of cells positively reacting with ATP1A1 antibody increased with the grade of GBM (Figure 1Bf and Table 1). We further evaluated ATP1A1 protein expression in GSCs and differentiated GBM cells from seven GBM tumors. We observed increased ATP1A1 expression in **five** of the seven GSC lines, and expression was not different from the differentiated GBM cells in **two** of the seven GSC lines (Figure 2A). We selected **two** patient-matched GSC lines (GBM GSCs1 and GBM GSCs2) for further research. Both GSC lines expressed the stemness markers nestin and SOX2 (Figure 2B) and GBM cells expressed the differentiation marker GFAP (Figure 2C). GSCs strongly reacted with ATP1A1 antibody (Figure 2D). Taken together, these results indicated that ATP1A1 is overexpressed in high-grade GBM and is generally upregulated in GSCs when compared with differentiated GBM cells.

**Table 1:** ATP1A1 expression in normal brain and glioma tissues of different grades.

**ATP1A1 Positivity**			
Tumor grade	+	++	+++
Normal brain	1	0	0
Grade I	1	0	0
Grade II	8	2	0
Grade III	4	11	5
GBM	2	3	10

*ATP1A1 positivity was defined by the percentage of cells that positively reacted with ATP1A1 antibody and was scored as follows: “+” 0–25%, “++” 26–75%, and “+++” 76–100%*.

### Knockdown of ATP1A1 Suppresses Proliferation and Survival in GSCs

**Figure 3:**
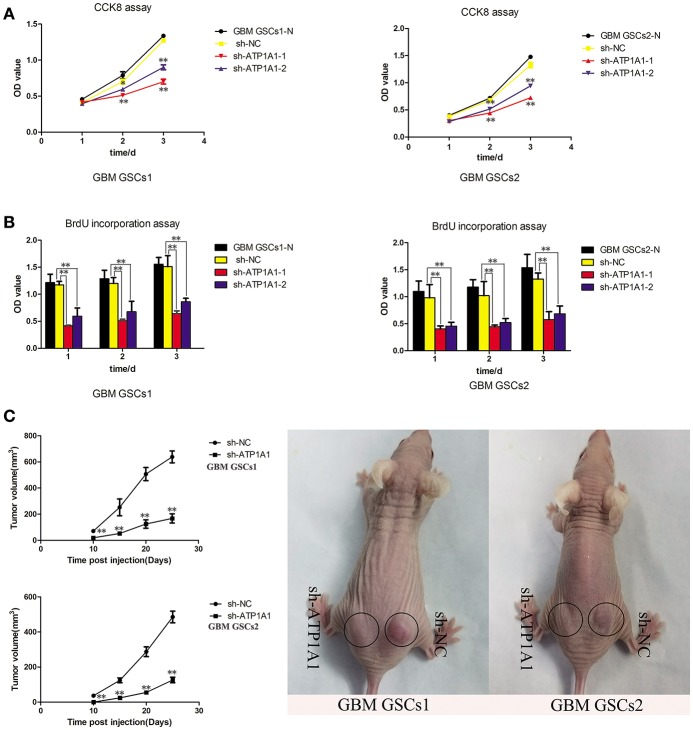
ATP1A1 knockdown decreases viability in GSC and tumor growth and proliferation. **(A)** CCK-8 and **(B)** BrdU incorporation assays were used to detect cell viability and proliferation in sh-NC- and sh-ATP1A1-transfected compared to non-transfected GSCs1 and GSCs2 cells. BrdU incorporation into DNA was measured by a colorimetric assay and absorbance was measured at 450 nm. **(C)** sh-ATP1A1- and sh-NC-transfected cells were injected subcutaneously into the flank of athymic nude mice (*n* = 5 mice/group). Tumor volume was monitored at the indicated times to plot tumor growth curves. The experiment was stopped at day 25, and the xenograft tumors were weighed. Data represent the mean ± SD, ^*^P < 0.05, ^**^P < 0.01.

To investigate the function of ATP1A1 in GSC growth further, we knocked down ATP1A1 expression in GBM GSCs1 and GBM GSCs2 by using shRNA. qPCR (Figure S1A) and western blot (Figure S1B) analyses showed that ATP1A1 expression was markedly decreased in cells transfected with sh-ATP1A1-1 or sh-ATP1A1-2 vector when compared with cells transfected with sh-NC vector and non-transfected cells (GBM GSCs1-N and GBM GSCs2-N). Compared to sh-ATP1A1-2, sh-ATP1A1-1 had higher knockdown efficiency. At 48 h and 72 h after transfection, proliferation and survival were significantly lower in sh-ATP1A1-transfected than in sh-NC-transfected cells (Figures 3A,B), with sh-ATP1A1-1 having the strongest effect. The tumor regrowth capacity of ATP1A1-knockdown GSCs was evaluated in xenograft model mice. The results showed that sh-ATP1A1 xenografts had significantly lower tumor volumes than sh-NC xenografts (Figure 3C). In addition, tumor weight was significantly lower in both sh-ATP1A1 groups than in the sh-NC group (GBM GSCs1: 0.62 and 0.264, respectively; GBM GSCs2: 0.08 vs. 0.024 g, respectively, *P* < 0.05 by Student's *t*-test). Thus, knockdown of ATP1A1 potentially suppresses the tumorigenicity of GSCs.

### ATP1A1 Knockdown Induces Cell Cycle Arrest and Enhances Apoptosis in GSCs

**Figure 4:**
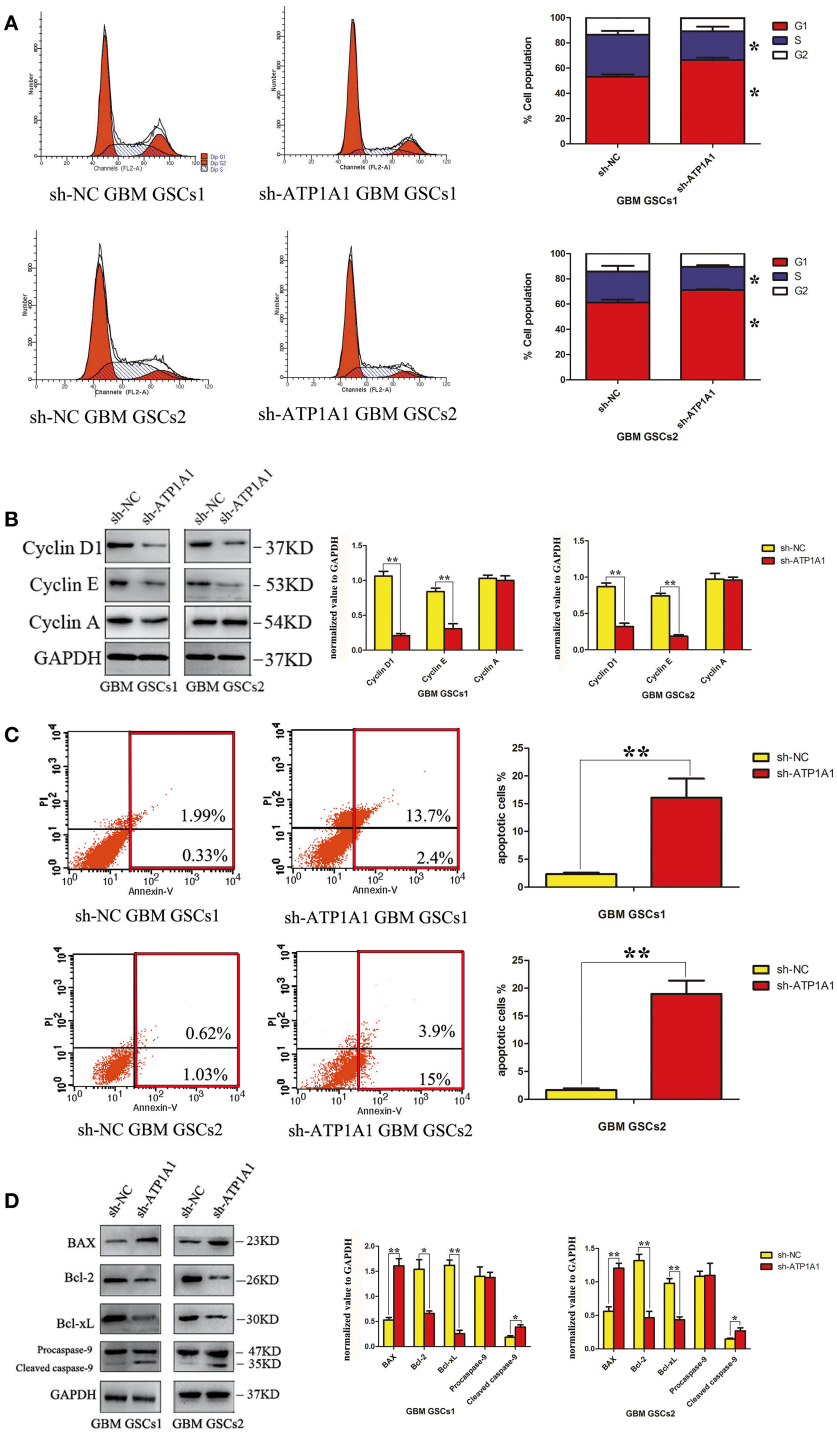
ATP1A1 knockdown in GSCs induces cell-cycle arrest and increases apoptosis. **(A)** The cellular DNA content was analyzed by flow cytometry. The histogram shows the percentage of cells in each phase of the cell cycle (right panel). The data represent means of five experiments. **(B)** Immunoblot analysis of key proteins in the G1, S, and G2–M cell-cycle phases in sh-ATP1A1- and sh-NC-transfected GSCs. **(C)** FACS analysis of cell apoptosis in sh-ATP1A1- and sh-NC-transfected GSCs. The cells were double stained with Annexin V and PI. Early apoptotic cells are in the lower right quadrant, late apoptotic cells are in the upper right quadrant. The histogram in the right panel shows average percentages of apoptotic cells which contain the early apoptotic cells and late apoptotic cells. **(D)** Immunoblot analysis of apoptosis-related proteins in sh-ATP1A1- and sh-NC-transfected cells. Experiments were repeated independently at least three times **(A–D)**. ^*^P < 0.05, ^**^P < 0.01.

To explore the mechanism by which ATP1A1 downregulation inhibits GSC proliferation, we analyzed specific phases of the cell cycle and tested key proteins in the G1, S, and G2–M cell-cycle phases (cyclin D1, cyclin E, and cyclin A, respectively) in sh-ATP1A1- and sh-NC-transfected cells by western blotting. sh-ATP1A1 knockdown resulted in an increase in cells in G1 phase and a concomitant decrease in cells in S phase, when compared with control cells (Figure 4A). Cyclin D1 and cyclin E were significantly decreased in sh-ATP1A1 GSCs, whereas cyclin A remained unchanged when compared with control cells (Figure 4B). These results indicated that ATP1A1 regulates G1/S phase transition. To evaluate the effect of ATP1A1 downregulation on GSC survival further, cell death was compared between control and ATP1A1 knockdown cells by flow-cytometric analysis. The fraction of apoptotic cells was larger in sh-ATP1A1-transfected than in sh-NC-transfected cells, and the total apoptotic rate was six times higher in ATP1A1-knockdown than in control cells (Figure 4C). We analyzed key apoptotic proteins to explore the underlying mechanisms. In ATP1A1-knockdown cells, Bax protein expression was significantly increased, whereas Bcl2 and Bcl-xL were remarkably reduced, and caspase 9 was activated. Two bands at 47 kDa (procaspase 9) and 35 kDa (cleaved caspase 9) could be observed, and cleaved caspase 9 abundance was increased in ATP1A1-knockdown cells (Figure 4D). Taken together, these results suggested ATP1A1 suppressed GSC proliferation and growth by inducing cell-cycle arrest and activating apoptosis signaling.

### Src May Be a Hub Regulating ATP1A1-Activated PI3K and MAPK Signaling

**Figure 5:**
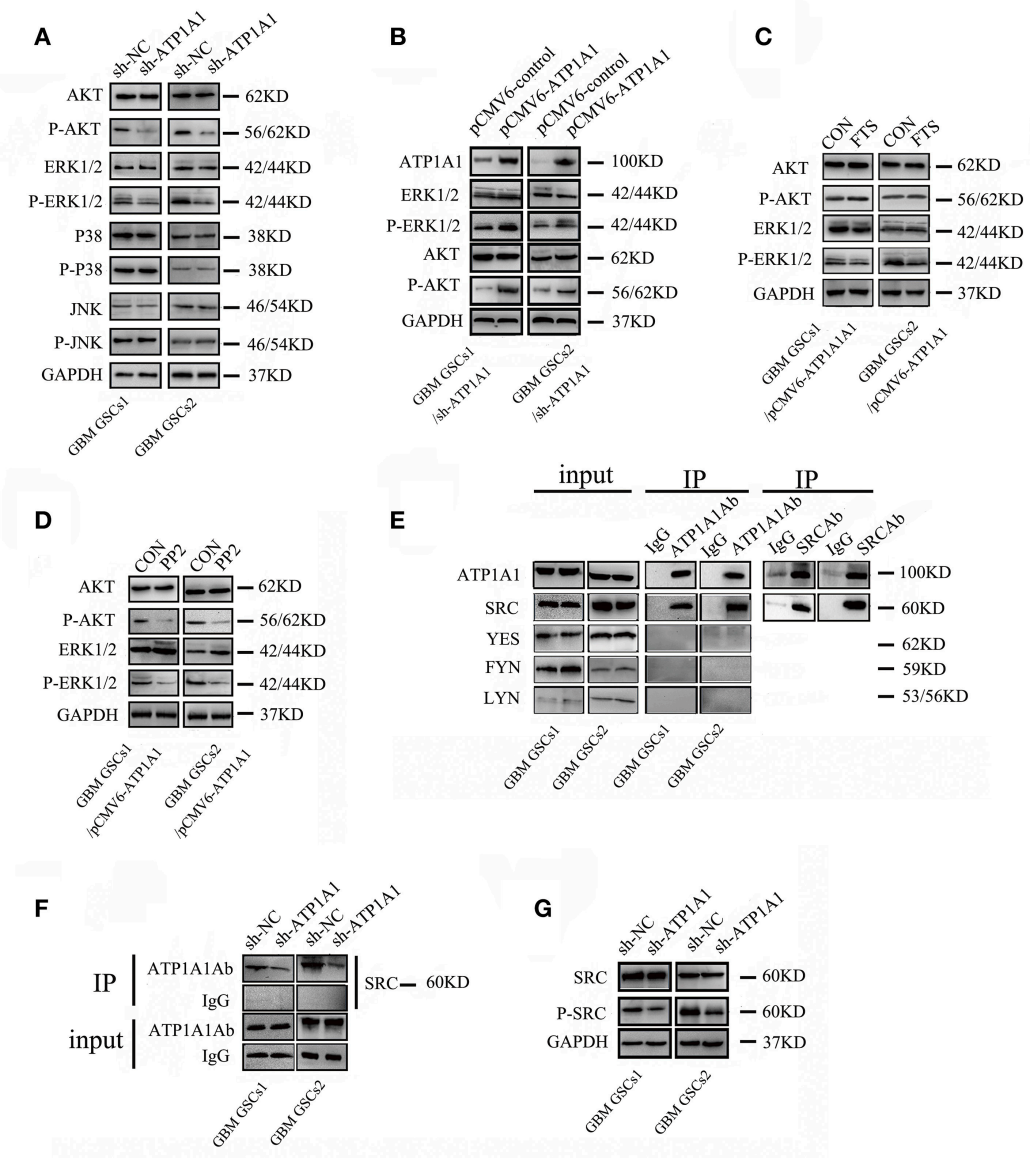
ATP1A1 enhances PI3K and MAPK/ERK signaling potentially via interacting with Src. **(A)** Effects of ATP1A1 on the phosphorylation status of ERK, JNK, p38, and AKT in GBM GSCs. **(B)** ATP1A1 cDNA was re-introduced into ATP1A1-knockdown cells and effectively restored signaling activation. **(C)** Effects of FTS on the phosphorylation status of ERK and AKT in GBM GSCs/pCMV-ATP1A1. **(D)** Effects of PP2 on the phosphorylation status of ERK and AKT in GBM GSCs/pCMV-ATP1A1. **(E)** ATP1A1/Src interaction was assayed by immunoprecipitation in GBM GSCs. **(F)** Changes in SRC expression in ATP1A1-silenced cells compared with control cells by immunoprecipitation with an anti-ATP1A1 antibody and IgG. To confirm that equal amounts of each cell extract were used for immunoprecipitation, whole-cell extracts were immunoblotted with anti-ATP1A1 and IgG (input). **(G)** Effects of ATP1A1 on the phosphorylation status of Src in sh-NC- and sh-ATP1A1-transfected GBM GSCs.

Our study indicated that ATP1A1 promotes survival and proliferation in GSCs. As the JNK, ERK, p38, and PI3K/AKT pathways are crucial in cell apoptosis, survival, and proliferation, we evaluated the roles of ATP1A1 in MAPK and PI3K/AKT pathway activation. ATP1A1 knockdown remarkably decreased ERK1/2 and AKT protein phosphorylation, without affecting total ERK1/2 and AKT levels. However, ATP1A1 knockdown did not affect the phosphorylation status of JNK or p38 (Figure 5A). Therefore, we speculated that ATP1A1 is upstream of AKT and ERK activation. Furthermore, we explored upstream events of ATP1A1 signal transduction to identify a hub for PI3K and MAPK pathway regulation. PI3K and MAPK pathways are important pathways downstream of Ras, and dysregulation of the Ras/MEK/ERK and Ras/PI3K/Akt pathways contributes to the proliferation of cancer-initiating cells and malignant cell growth (20). Thus, we first focused on Ras. Introduction of ATP1A1 cDNA into ATP1A1-knockdown cells (referred to as GSCS/pCMV-ATP1A1) resulted in the recovery of AKT and ERK signaling activation (Figure 5B), proliferation, and survival (Figure S2). FTS is an effective Ras inhibitor without toxicity that destabilizes the interaction of Ras with the cell membrane and inhibits the activation of Ras-dependent signaling. As expected, in the FTS treatment group, ERK phosphorylation was suppressed in GBM GSCs1/pCMV-ATP1A1 and GBM GSCS2/pCMV-ATP1A1; however, AKT phosphorylation was not affected (Figure 5C). These results indicated that ATP1A1 regulated AKT activation via another pathway and Ras is not a hub in MAPK/ERK and PI3K pathway activation by ATP1A1.

SFKs interact with the α-subunit of Na^+^/K^+^-ATPase to initiate signaling. There are nine members in the family, four of which (Src, FYN, YES, and LYN) are expressed in human gliomas (21). Activation of Src-MAPK/AKT signaling helps cancer cells to survive and proliferate (17). Therefore, we next focused on Src, using the Src inhibitor PP2. In the PP2 treatment group, ERK and AKT phosphorylation was almost completely abolished in GBM GSCs1/pCMV-ATP1A1 and GBM GSCS2/pCMV-ATP1A1 (Figure 5D). These results indicated that in our GSC model, the re-introduction of ATP1A1 failed to recover ERK and AKT phosphorylation in PP2-treated cells, and we speculated Src maybe a hub for PI3K and MAPK pathway activation by ATP1A1. Next, we clarified whether Src interacts with ATP1A1 in our GSC models. We immunoprecipitated cell lysates with an anti-ATP1A1 or anti-Src antibody and found that endogenous ATP1A1 immunoprecipitated with Src (Figure 5E). The amount of Src protein co-immunoprecipitated with anti-ATP1A1 antibody in ATP1A1-knockdown cells was dramatically less than that in control cells (Figure 5F). In addition, we validated the activation of Src in our sh-ATP1A1 GSC models by measuring its specific phosphorylation at Y416. The data showed that knockdown of ATP1A1 suppressed Src protein (pY416) phosphorylation, without affecting the total Src protein amount (Figure 5G). These data suggested that ATP1A1 may interact with Src to activate tyrosine phosphorylation of Src.

### ATP1A1 Enhances Proliferation and Survival Likely via an Src-Dependent Mechanism

**Figure 6:**
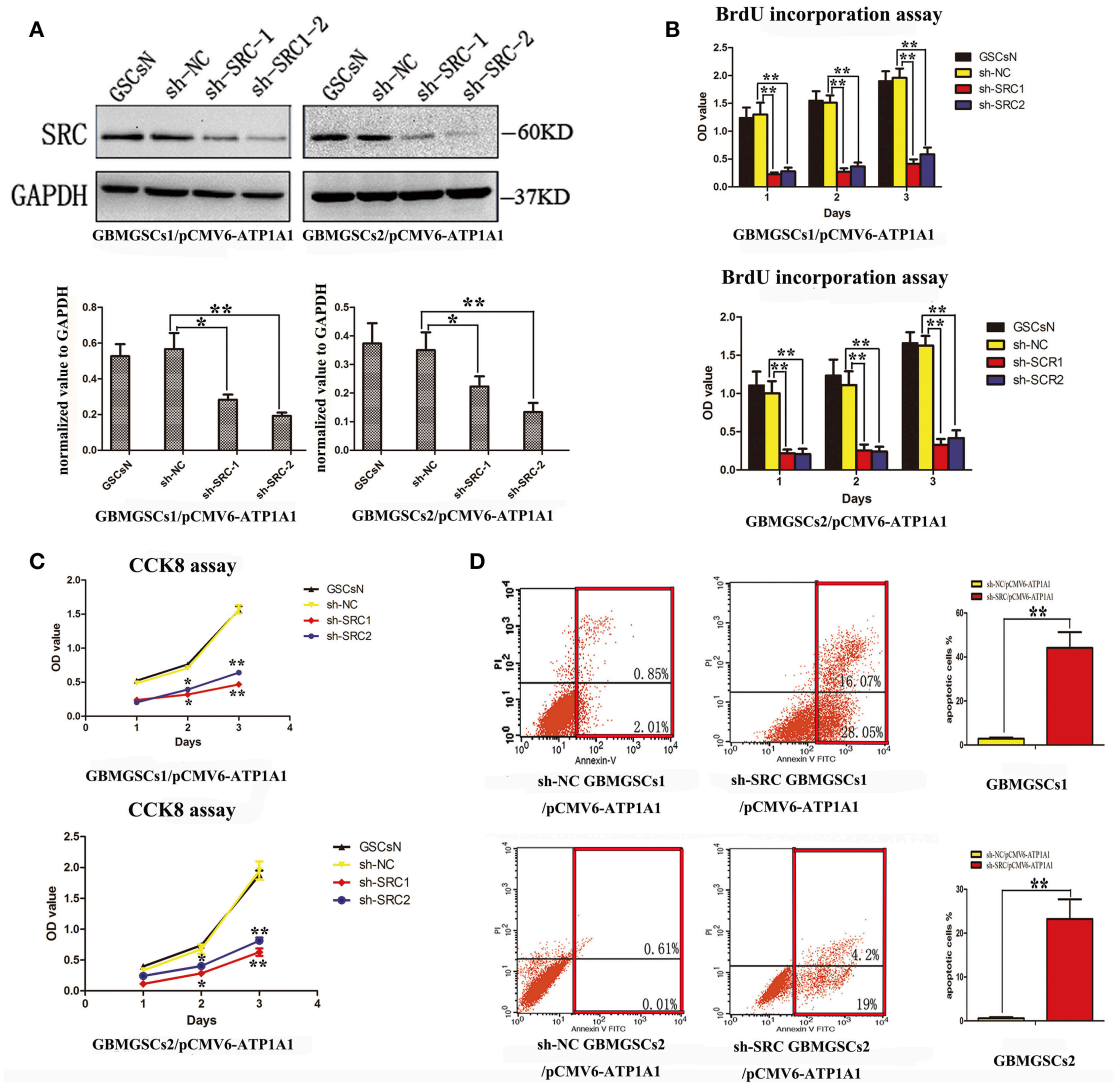
ATP1A1 promotes GSC proliferation and survival in a Src-dependent manner. **(A)** Src knockdown efficiency in GBM GSCs/pCMV6-ATP1A1 was confirmed by western blotting. **(B)** Cell growth curves of BrdU incorporation assays and **(C)** CCK-8 assays in GBM GSCs/pCMV6-ATP1A1 cells with Src knockdown. Data were collected at 24, 48, and 72 h. **(D)** Flow-cytometric analysis of GBM GSCs/pCMV6-ATP1A1 cells after Src knockdown. The cells were double stained with Annexin V and PI. Early apoptotic cells are in the lower right quadrant, late apoptotic cells are in the upper right quadrant. The histogram in the right panel shows average percentages of apoptotic cells which contain the early apoptotic cells and late apoptotic cells. Error bars represent the SD, ^*^P < 0.05, ^**^P < 0.01.

To further clarify whether knockdown of ATP1A1 resulted in suppression of GSC proliferation and growth via Src, we suppressed Src expression in sh-Src GSCs/pCMV6-ATP1A1 models and then determined the cell proliferation and survival capacities. The knockdown efficiency was confirmed by western blotting (Figure 6A). Like sh-ATP1A1 cells, sh-Src GSCs/pCMV6-ATP1A1 showed significantly suppressed proliferation (Figure 6B) and survival (Figure 6C), and increased apoptosis (Figure 6D). These data demonstrated that knockdown of Src inhibited cell proliferation and survival, which were restored by rescuing the level of ATP1A1, in our GSC models. Taken together, our data suggested that ATP1A1 promotes Src phosphorylation to activate MAPK/ERK and PI3K/AKT signaling pathways by interacting directly or indirectly with Src, thereby promoting growth and survival in GSCs.

## Discussion

Glioblastomas are one of the most lethal tumors, and GSCs pose a new challenge in the treatment of glioblastomas (22). GSCs are a source of gliomas that maintain the growth of tumors and are contribute to glioma recurrence (23). Compared to non-stem tumor cells, GSCs express specific markers which play an important role in maintaining the biological characteristics of GSCs (24). To screen for specific molecules expressed in GSCs, we previously performed a proteomics assay comparing GSCs and differentiated glioma cells, and we found that ATP1A1 is overexpressed in GSCs (7). Motivated by this finding, we determined the role of ATP1A1 in the malignant phenotype and pathogenesis of GSCs. The results indicated that ATP1A1 is preferentially expressed in high-grade GBM and GSCs, and knockdown of ATP1A1 effectively inhibited GSC proliferation and growth by suppressed Src-mediated activating phosphorylation of PI3K and MAPK. In this study, we confirmed that ATP1A1 is involved in regulating progression of GSCs development, and our findings may offer a novel candidate for developing targeted therapeutic strategies for GSCs.

Aberrant expression and activity of Na^+^/K^+^-ATPases have been thought to contribute to the development and progression of different types of cancer (25). ATP1A1 reportedly is overexpressed in non-small cell lung cancer, melanomas, and glioblastomas (20, 26). In our study, ATP1A1 was generally overexpressed in GSCs, and the expression level of ATP1A1 positively correlated with the pathological grade of human glioma, while the higher grade glioma is closely related to the worse prognosis of patients. Na^+^/K^+^-ATPase has been reported to promote cancer initiation, development, and proliferation by regulating various cell-cycle and apoptosis pathways (27, 28). In this study, knockdown of ATP1A1 effectively inhibited cell proliferation and growth, and decreased AKT and ERK1/2 phosphorylation in our GSC models, while re-introduction of ATP1A1 restored cell function. Thus, we suggest that ATP1A1 is involved in the biological progression of GSCs and activates downstream AKT and ERK key survival pathways to improve cell growth and proliferation.

The three classic Ras pathways are the MAPK, PI3K/AKT, and Ral-GDS/Ral pathways, which control cell proliferation, survival, and motility and cytoskeletal arrangements, respectively (29–31). Ras is continuously activated in human malignant astrocytomas (32), and activated Ras promotes PI3K/AKT and ERK signaling activation to regulate proliferation, survival, and cell migration in lung cancer and oral carcinoma (33, 34). Accordingly, activated Ras/Raf/MEK/ERK and Ras/PI3K/AKT signaling may be critical for proliferation and survival in glioma (35). Our results showed that the PI3K pathway was activated by ATP1A1 when Ras-mediated AKT and ERK signaling transduction were inhibited by FTS, which indicated that Ras is not solely involved in ATP1A1-depended activation of the PI3K pathway in our GSC models. Src is a membrane-associated non-receptor tyrosine kinase. Studies have shown that treatment of glioma cells with a specific Src inhibitor (PP2 or dasatinib) suppresses migration and proliferation (36–38). Elevated Src kinase activity affects tumor progression by activating MAPK and PI3K/AKT pathways (39, 40). Src has two main phosphorylation sites; tyrosine 416, phosphorylation of which results in Src activation, and tyrosine 527, phosphorylation of which results in inhibition of Src activity (39–41). Similarly, in our study, ATP1A1 might improve cell proliferation and growth through activating Src-mediated MAPK and PI3K signaling pathways, and we confirmed that knockdown of ATP1A1 decreased Src phosphorylation at Y416. Interestingly, there is currently controversy about the direct interaction between ATP1A1 and Src. Src reportedly can bind to intracellular domains of the Na^+^/K^+^-ATPase α-subunit and thus regulate downstream proteins and cell functions (42). In contrast, it has been also reported that ATP1A1 inhibits Src activation by binding to Src (43), which seems at odds with our result. However, Tian et al. (20) and Ye et al. (18) showed that α1 interacts directly with Src, and that Src is activated by binding of its SH2 domain to the second cytosolic domain of α1 and is inactivated by binding of its kinase domain to the nucleotide-binding domain of α1. Therefore, in our study, we speculate that ATP1A1 might affect the normal conformation transition to regulate Src kinase activation. In future, the exact mechanism of and direct evidence for ATP1A1-Src interaction in our GSC models should be investigated, and our findings should be validated in more GSCs.

In conclusion, our study demonstrated for the first time that ATP1A1 supports proliferation and survival in primary human GSCs and revealed that Src may be a key molecule involved in ATP1A1-dependent PI3K and MAPK pathway activation. Our findings will aid in further basic research and may provide a novel approach to sabotaging GSCs and glioma therapies based on stemness-related targets.

## Ethics Statement

In this study, all procedures were approved by Ethics Review Board of Chongqing Medical University (Chongqing, China), and gained the written consent of all patients.

## Author Contributions

SW and SC designed and conducted the research, analyzed the data, and critically reviewed the manuscript. YY, CC, and JD performed the experiments, analyzed data, and drafted the original manuscript. GH, HZ, LJ, and RX participated in data analysis. All authors read and approved the final manuscript.

### Conflict of Interest Statement

The authors declare that the research was conducted in the absence of any commercial or financial relationships that could be construed as a potential conflict of interest.

## References

[1] Ohgaki H Dessen P Jourde B Horstmann S Nishikawa T Di Patre PL. Genetic pathways to glioblastoma: a population based study. Cancer Res. (2004) 64:6892–9. 10.1158/0008-5472.CAN-04-1337 15466178

[2] Reardon DA Rich JN Friedman HS Bigner DD. Recent advances in the treatment of malignant astrocytoma. J Clin Oncol. (2006) 24:1253–65. 10.1200/JCO.2005.04.5302 16525180

[3] Reardon DA Wen PY Glioma in 2014: unravelling tumor heterogeneity-implications for therapy. Nat Rev Clin Oncol. (2015) 12:69–70. 10.1038/nrclinonc.2014.223 25560529

[4] Hsu HS Lin JH Huang WC Hsu TW Su K Chiou SH. Chemoresistance of lung cancer stemlike cells depends on activation of Hsp27. Cancer. (2011) 117:1516–28. 10.1002/cncr.25599 21425153

[5] Vermeulen L Sprick MR Kemper K Stassi G Medema JP. Cancer stem cells-old concepts, new insights. Cell Death Differ. (2008) 15:947–58. 10.1038/cdd.2008.20 18259194

[6] Li Z Bao S Wu Q Wang H Eyler C Sathornsumetee S. Hypoxia-inducible factors regulate tumorigenic capacity of glioma stem cells. Cancer Cell. (2009) 15:501–13. 10.1016/j.ccr.2009.03.018 19477429 PMC2693960

[7] Chen S Zhao H Deng J Liao P Xu Z Cheng Y. Comparative proteomics of glioma stem cells and differentiated tumor cells identifies S100A9 as a potential therapeutic target. J Cell Biochem. (2013) 114:2795–808. 10.1002/jcb.24626 23836528

[8] Lan YL Yu ZL Lou JC Ma XC Zhang B. Update on the effects of the sodium pump α1 subunit on human glioblastoma: from the laboratory to the clinic. Expert Opin Investig Drugs. (2018) 27:753–63. 10.1080/13543784.2018.1512582 30130132

[9] Kaplan JH. Biochemistry of Na,K-ATPase. Annu Rev Biochem. (2002) 71:511–35. 10.1146/annurev.biochem.71.102201.141218 12045105

[10] Lefranc F Kiss R. The sodium pump α1 subunit as a potential target to combat apoptosis-resistant glioblastomas. Neoplasia. (2008) 10:198–206. 10.1593/neo.07928 18323016 PMC2259449

[11] Lefranc F Mijatovic T Kondo Y Sauvage S Roland I Debeir O. Targeting the alpha 1 subunit of the sodium pump to combat glioblastoma cells. Neurosurgery. (2008) 62:211–21; discussion 221–2. 10.1227/01.NEU.0000311080.43024.0E 18300910

[12] Lan YL Wang X Lou JC Xing JS Yu ZL Wang H Bufalin inhibits glioblastoma growth by promoting proteasomal degradation of the Na^+^/K^+^-ATPase α1 subunit. Biomed Pharmacother. (2018) 103:204–15. 10.1016/j.biopha.2018.04.030 29653366

[13] Sayed M Drummond CA Evans KL Haller ST Liu J Xie Z. Effects of Na/K-ATPase and its ligands on bone marrow stromal cell differentiation. Stem Cell Res. (2014) 13:12–23. 10.1016/j.scr.2014.04.002 24793006 PMC4090276

[14] Lee YK Ng KM Lai WH Man C Lieu DK Lau CP. Ouabain facilitates cardiac differentiation of mouse embryonic stem cells through ERK1/2 pathway. Acta Pharmacol Sin. (2011) 32:52–61. 10.1038/aps.2010.188 21151160 PMC4003319

[15] Durlacher CT Chow K Chen XW He ZX Zhang X Yang T. Targeting Na^+^/K^+^ -translocating adenosine triphosphatase in cancer treatment. Clin Exp Pharmacol Physiol. (2015) 42:427–43. 10.1111/1440-1681.12385 25739707

[16] Patel A Sabbineni H Clarke A Somanath PR. Novel roles of Src in cancer cell epithelial-to-mesenchymal transition, vascular permeability, microinvasion and metastasis. Life Sci. (2016) 157:52–61. 10.1016/j.lfs.2016.05.036 27245276 PMC4956571

[17] Qi S Feng Z Li Q Qi Z Zhang Y. Inhibition of ROS-mediated activation Src-MAPK/AKT signaling by orientin alleviates H_2_O_2_-induced apoptosis in PC12 cells. Drug Des Devel Ther. (2018) 12:3973–84. 10.2147/DDDT.S178217 30510405 PMC6248275

[18] Ye Q Li Z Tian J Xie JX Liu L Xie Z. Identification of a potential receptor that couples ion transport to protein kinase activity. J Biol Chem. (2011) 286:6225–32. 10.1074/jbc.M110.202051 21189264 PMC3057788

[19] Pollard SM Yoshikawa K Clarke ID Danovi D Stricker S Russell R. Glioma stem cell lines expanded in adherent culture have tumor-specific phenotypes and are suitable for chemical and genetic screens. Cell Stem Cell. (2009) 4:568–80. 10.1016/j.stem.2009.03.014 19497285

[20] Tian J Cai T Yuan Z Wang H Liu L Haas M Binding of Src to Na^+^/K^+^-ATPase forms a functional signaling complex. Mol Biol Cell. (2006) 17:317–26. 10.1091/mbc.e05-08-0735 16267270 PMC1345669

[21] Han X Zhang W Yang X Wheeler CG Langford CP Wu L. The role of Src family kinases in growth and migration of glioma stem cells. Int J Oncol. (2014) 45:302–10. 10.3892/ijo.2014.2432 24819299 PMC4079155

[22] Mehta S. The role of microenvironment in the homing, maintenance, and release of glioma stem-like cells. Front Oncol. (2018) 8:7. 10.3389/fonc.2018.00007 29441325 PMC5797621

[23] Schonberg DL Lubelski D Miller TE Rich JN. Brain tumor stem cells: molecular characteristics and their impact on therapy. Mol Aspects Med. (2014) 39:82–101. 10.1016/j.mam.2013.06.004 23831316 PMC3866208

[24] Zhou D Alver BM Li S Hlady RA Thompson JJ Schroeder MA. Distinctive epigenomes characterize glioma stem cells and their response to differentiation cues. Genome Biol. (2018) 19:43. 10.1186/s13059-018-1420-6 29587824 PMC5872397

[25] Uusi-Rauva K Luiro K Tanhuanpää K Kopra O Martín-Vasallo P Kyttälä A. Novel interactions of CLN3 protein link Batten disease to dysregulation of fodrin-Na^+^, K^+^ ATPase complex. Exp Cell Res. (2008) 314:2895–905. 10.1016/j.yexcr.2008.06.016 18621045

[26] Pore MM Hiltermann TJ Kruyt FA. Targeting apoptosis pathways in lung cancer. Cancer Lett. (2013) 332:359–68. 10.1016/j.canlet.2010.09.012 20974517

[27] Babula P Masarik M Adam V Provaznik I Kizek R From Na^+^/K^+^-ATPase and cardiac glycosides to cytotoxicity and cancer treatment. Anticancer Agents Med Chem. (2013) 13:1069–87. 10.2174/18715206113139990304 23537048

[28] Prassas I Diamandis EP. Novel therapeutic applications of cardiac glycosides. Nat Rev Drug Discov. (2008) 7:926–35. 10.1038/nrd2682 18948999

[29] Stout MC Asiimwe E Birkenstamm JR Kim SY Campbell PM. Analyzing Ras-associated cell proliferation signaling. Methods Mol Biol. (2014) 1170:393–409. 10.1007/978-1-4939-0888-2_21 24906326

[30] Rampias T Giagini A Siolos S Matsuzaki H Sasaki C Scorilas A. RAS/PI3K crosstalk and cetuximab resistance in head and neck squamous cell carcinoma. Clin Cancer Res. (2014) 20:2933–46. 10.1158/1078-0432.CCR-13-2721 24696319

[31] Cooper JM Bodemann BO White MA. The RalGEF/Ral pathway: evaluating an intervention opportunity for Ras cancers. Enzymes. (2013) 34(Pt B):137–56. 10.1016/B978-0-12-420146-0.00006-8 25034103

[32] Guha A Feldkamp MM Lau N Boss G Pawson A. Proliferation of human malignant astrocytomas is dependent on Ras activation. Oncogene. (1997) 15:2755–65. 9419966 10.1038/sj.onc.1201455

[33] James MA Vikis HG Tate E Rymaszewski AL You M. CRR9/CLPTM1L regulates cell survival signaling and is required for Ras transformation and lung tumorigenesis. Cancer Res. (2014) 74:1116–27. 10.1158/0008-5472.CAN-13-1617 24366883 PMC6005686

[34] Wang H Wu Q Liu Z Luo X Fan Y Liu Y Downregulation of FAP suppresses cell proliferation and metastasis through PTEN/PI3K/AKT andRas-ERK signaling in oral squamous cell carcinoma. Cell Death Dis. (2014) 5:e1155 10.1038/cddis.2014.122 24722280 PMC5424105

[35] Breunig JJ Levy R Antonuk CD Molina J Dutra-Clarke M Park H. Ets factors regulate neural stem cell depletion and gliogenesis in Ras pathway glioma. Cell Rep. (2015) 12:258–71. 10.1016/j.celrep.2016.12.026 26146073

[36] Liu J Xie ZJ. The sodium pump and cardiotonic steroids-induced signal transduction protein kinases and calcium-signaling microdomain in regulation of transporter trafficking. Biochim Biophys Acta. (2010) 1802:1237–45. 10.1016/j.bbadis.2010.01.013 20144708 PMC5375027

[37] Schlessinger J. New roles for Src kinases in control of cell survival and angiogenesis. Cell. (2000) 100:293–6. 10.1016/S0092-8674(00)80664-9 10676810

[38] Xu J Wu RC O'Malley BW. Normal and cancer-related functions of the p160 steroid receptor co-activator (SRC) family. Nat Rev Cancer. (2009) 9:615–30. 10.1038/nrc2695 19701241 PMC2908510

[39] Tamiya S Okafor MC Delamere NA. Purinergic agonists stimulate lens Na-K-ATPase-mediated transport via a Src tyrosine kinase-dependent pathway. Am J Physiol Cell Physiol. (2007) 293:C790–6. 10.1152/ajpcell.00579.2006 17522142

[40] Tsang JL Jia SH Parodo J Plant P Lodyga M Charbonney E. Tyrosine phosphorylation of caspase-8 abrogates its apoptotic activity and promotes activation of c-Src. PLoS ONE. (2016) 11:e0153946. 10.1371/journal.pone.0153946 27101103 PMC4839753

[41] Roskoski R. Src protein-tyrosine kinase structure and regulation. Biochem Biophys Res Commun. (2004) 324:1155–64. 10.1016/j.bbrc.2004.09.171 15504335

[42] Wu J Akkuratov EE Bai Y Gaskill CM Askari A Liu L Cell signaling associated with Na^+^/K^+^-ATPase: activation of phosphatidylinositide 3-kinase IA/Akt by ouabain is independent of Src. Biochemistry. (2013) 52:9059–67. 10.1021/bi4011804 24266852 PMC3868411

[43] Li Z Cai T Tian J Xie JX Zhao X Liu L. Na/K-ATPase-derived peptide Src inhibitor, antagonizes ouabain-activated signaltransduction in cultured cells. J Biol Chem. (2009) 284:21066–76. 10.1074/jbc.M109.013821 19506077 PMC2742871

