# Supplementary material for: ATP1A1 Integrates AKT and ERK Signaling via Potential Interaction With Src to Promote Growth and Survival in Glioma Stem Cells
Source: Front Oncol. 2019 Apr 30;9:320. doi: 10.3389/fonc.2019.00320 (PMC6503087; doi:10.3389/fonc.2019.00320)
Supplement: Supplementary file 1 [file Data_Sheet_1.docx]

Supplementary Figures with legends

**

**

**Figure S1. Knockdown of ATP1A1 expression in GSCs**. GBM GSCs1 and GBM GSCs2 were transfected with vectors expressing different shRNAs silencing ATP1A1 (sh-ATP1A1-1, sh-ATP1A1-2, and sh- ATP1A1-3) or negative control vector (sh-NC). Knockdown efficiencies of shRNAs were assessed by qPCR (A) and western blot (B) analyses. Error bars represent the SD, **P* < 0.05, ***P* < 0.01).


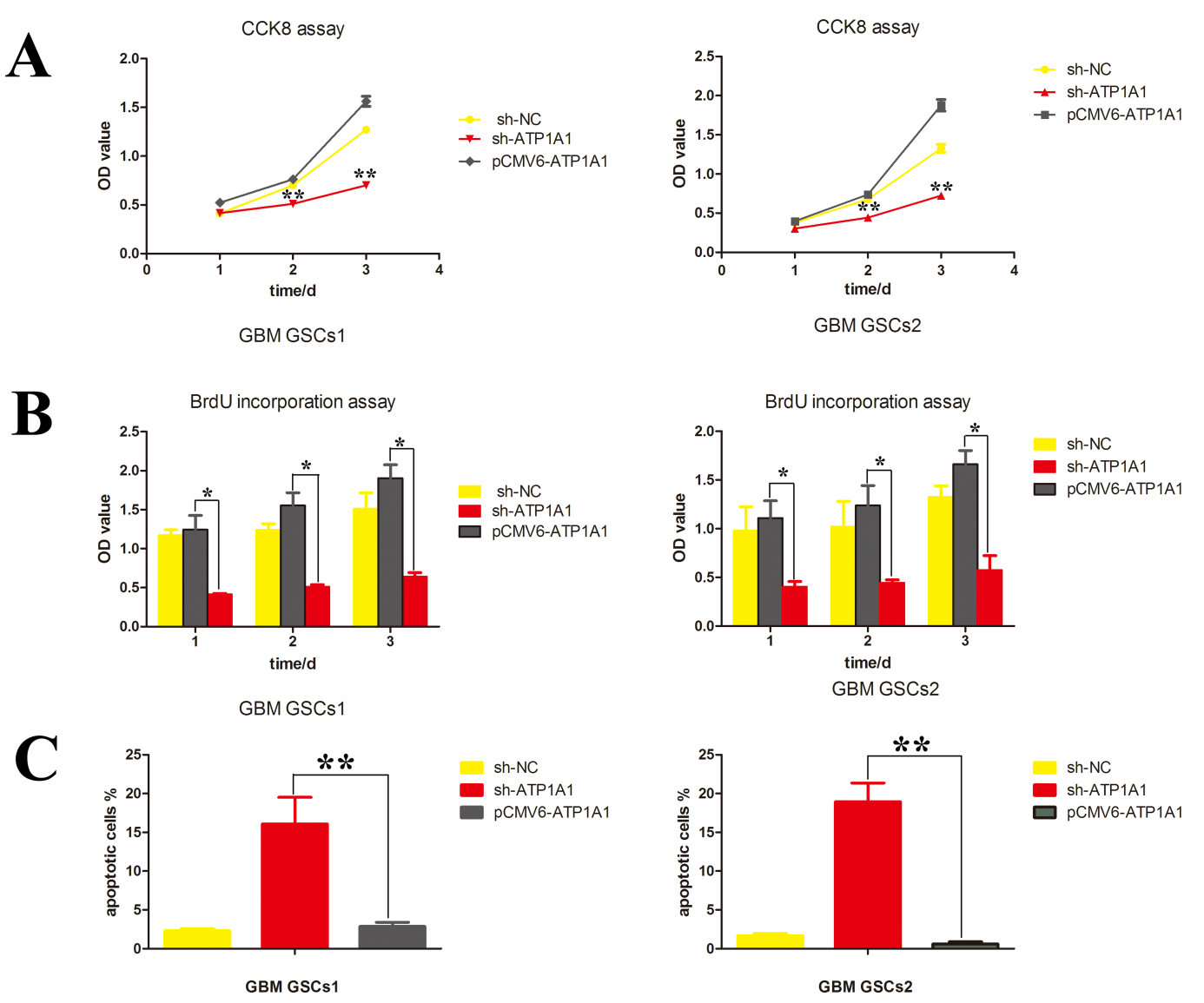


**Figure S2. Re-transduction of ATP1A1 in ATP1A1-knockdown cells restores proliferation and viability.** ATP1A1-knockdown cells (sh-ATP1A1 cells) were transfected with ATP1A1 cDNA to recover ATP1A1 expression. Proliferation and survival in the cells were examined by CCK-8 (A), BrdU incorporation assays (B) and flow-cytometric analysis (C).
