# Supplementary material for: ATP1A1 Integrates AKT and ERK Signaling via Potential Interaction With Src to Promote Growth and Survival in Glioma Stem Cells
Source: Front Oncol. 2019 Apr 30;9:320. doi: 10.3389/fonc.2019.00320 (PMC6503087; doi:10.3389/fonc.2019.00320)
Supplement: Supplementary file 2 [file Data_Sheet_2.docx]

Supplementary Material

# Supplementary Materials and Methods

## Western blotting

Cell lysates were prepared in lysis buffer (20 mM HEPES pH 7.4, 1% Nonidet P-40, 150 mM NaCl, 5 mM MgCl_2_, and 10% glycerol). The protein samples (approximately 30 µg) were separated by sodium dodecyl sulfate polyacrylamide gel electrophoresis and transferred to polyvinylidene fluoride membranes. The membranes were blocked for 1 h with 1% bovine serum albumin in TBS-T buffer (20 mmol/l Tris pH 7.6, 100 mmol/L NaCl_2_, and 0.5% Tween-20), followed by overnight incubation with the primary antibodies at 4°C. Primary antibodies targeting ATP1A1 (#71638, 1:500 dilution, Santa Cruz, CA, USA), pERK1/2 (#9101, 1:2000 dilution, Cell Signaling Technology, MA, USA), ERK1/2 (#9102, 1:1000 dilution, Cell Signaling Technology, MA, USA), Akt (#9272, 1:1000 dilution, Cell Signaling Technology, MA, USA), pAkt (#9611, 1:2000 dilution, Cell Signaling Technology, MA, USA), p38 (#9212, 1:1000 dilution, Cell Signaling Technology, MA, USA), pp38 (#9216, 1:2000 dilution, Cell Signaling Technology, MA, USA), JNK (#9252, 1:1000 dilution, Cell Signaling Technology, MA, USA), pJNK (#9255, 1:2000 dilution, Cell Signaling Technology, MA, USA) Src and pSrc (pY416) (#9252, 1:1,000 dilution and #6943, 1:2,000 dilution, Cell Signaling Technology, MA, USA), YES (#3201, 1:1000 dilution, Cell Signaling Technology, MA, USA), FYN (#4023, 1:1000 dilution, Cell Signaling Technology, MA, USA), LYN (#4576, 1:1000 dilution, Cell Signaling Technology, MA, USA), LCK (#2714, 1:1000 dilution, Cell Signaling Technology, MA, USA), Cyclin D1 (#70899, 1:500 dilution, Santa Cruz, CA, USA), Cyclin E (#377100, 1:500 dilution, Santa Cruz, CA, USA), Cyclin A (#239, 1:500 dilution, Santa Cruz, CA, USA), Bax (#20067, 1:500 dilution, Santa Cruz, CA, USA), Bcl2 (#509, 1:500 dilution, Santa Cruz, CA, USA), Bcl-XL (#8392, 1:500 dilution, Santa Cruz, CA, USA), Caspase 9 (#133109, 1:500 dilution, Santa Cruz, CA, USA) were used. The secondary antibodies were purchased from Abcam (Cambridge, MA, USA). The protein bands were visualized by an ECL detection system, and protein band densities were analyzed using ImageJ software. All western blot analyses were repeated at least thrice. The relative changes in protein levels were determined by densitometry with normalization to GAPDH. Data are presented as means ± SD.

## Immunocytochemistry

Cells (2.5 × 10^5^) were plated on coverslips precoated with laminin (Invitrogen) and polylysine (Sigma) (5 µg/ml each). They were fixed in 4% paraformaldehyde for 30 min, then washed three times in phosphate-buffered saline (PBS) and blocked with 5% goat serum at 37°C for 30 min. Antibodies against nestin (#71665, 1:100 dilution, Santa Cruz, CA, USA),SOX2 (#3579, 1:100 dilution, Cell Signaling Technology, MA, USA), GFAP (1: 50, CusAb, College Park, MD, USA) and ATP1A1 (#71638, 1:100, Santa Cruz, CA, USA)were used. Stained dells were first permeabilized in 0.5% Triton X-100 (Sigma) in 10% fetal bovine serum in PBS for 20 min at 4°C, washed three times, and labeled at 4°C overnight. Cells were washed three times for 10 min each then incubated with Cy3-conjugated goat anti-mouseantibody (1:100 dilution, BioLegend, San Diego, CA, USA) and FITC-conjugated goat anti-mouse antibody (1:100, BioLegend)at 37°C for 1 h. Cells were washed to remove unbound secondary antibody, mounted with 4′,6′-diamidino-2-phenylindole for 5 min at room temperature, and then detected with a fluorescence microscope (Nikon TE2000-U, Tokyo, Japan).

## BrdU incorporation and CCK-8 assays

Cell proliferation was assayed using a BrdU incorporation kit (Roche Applied Science, Basel, Switzerland), according to the manufacturer’s protocol. Briefly, equal numbers of cells were seeded in 96-well plates at 5,000 cells/well. Following incubation, the cells were labeled with BrdU for 2 h and the optical density at 450 nm was measured. A CCK-8 assay (Sigma, St Louis, MO, USA) was used to measure cell viability at different time points. Briefly, cells were seeded in 96-well plates at 5,000 cells/well, and following incubation with CCK-8 dye, the absorbance at 490 nm was measured. For both BrdU and CCK-8 assays, cells of each group were seeded in five wells each, and the experiment was repeated at least three times.

## Flow-cytometric analysis of cell cycle and apoptosis

Cells were harvested and fixed in 5 ml of ice-cold 70% ethanol at 4°C. Cells were pelleted by centrifugation and resuspended in 400 ml of PBS, 50 ml of propidium iodide (PI) solution (0.6 mM), and 50 ml of RNase A (1 mg/ml). After 30 min, the cells were analyzed for DNA content using a FACS flow cytometer. Fluorescence from the PI-DNA complex was estimated for a minimum of 10,000 cells per sample and analyzed with CellQuest Pro software. To determine apoptosis, cells were harvested and incubated with reagents from the Annexin V-PI apoptosis kit (BioVision, CA, USA), according to manufacturer’s protocol.

## CSC tumorigenicity assays in athymic nude mice

Animal studies to assess CSC tumorigenicity *in vivo* were conducted according to the Chongqing Medical University Institutional guidelines. Six to eight-week-old female athymic nude mice were obtained from Chongqing Medical University and were housed in a specific pathogen-free environment at Chongqing Medical University. GSCs transfected with lentivirus expressing non-targeting shRNA (sh-NC) or ATP1A1-targeting shRNA (sh-ATP1A1) were suspended in PBS and injected subcutaneously into the flank of athymic nude mice (2×10^6^ cells/mouse and n = 5 mice/group). The mean tumor volume at the indicated time was plotted to monitor tumor growth. Tumor volume was calculated according to the following formula: length × (width)^2^/2. The mice were sacrificed on day 25, and the xenograft tumors were dissected and weighed.

## Immunohistochemical analysis of tissue microarrays

Tissue samples were provided as microarrays (US Biomax, Rockville, MD, USA catalog Nos. GL722 and GL807). Fifteen of the 50 samples had a GBM (age: 36 ± 17 years; 6 females and 9 males), 20 samples had a WHO grade III astrocytoma (age: 46 ± 11 years; 7 females and 12 males), 10 samples had a WHO grade II astrocytoma (age: 42 ±12 years; 5 females and 8 males), five samples had a WHO grade I astrocytoma (age: 42 ± 10 years; 1 female and 2 males), and eight samples were from healthy subjects (age: 41 ± 10 years; 4 females and 4 males). The tissue samples originated from different donors, and each sample had at least two replicates. The glioma tissue sections were from the tumor areas and did not include adjacent normal tissues.

Immunohistochemical staining was performed using the tissue microarrays. The paraffin-embedded 5-µm arrays were dewaxed in xylene for 10 min and rehydrated through a series of alcohol solutions (100% ethanol twice, 90% ethanol, and 70% ethanol for 5 min each) and finally, in water. Antigen retrieval was achieved by boiling the arrays in citrate buffer at pH 6.0. Endogenous peroxidase activity was blocked using 6% H_2_O_2_. The tissue microarrays were blocked with 2% normal goat serum and incubated with rabbit anti-human ATP1A1 monoclonal antibody overnight at 4°C. Immunodetection was carried out using the EliVision^TM^ plus system (Sigma, St. Louis, MO, USA), according to the manufacturer’s instructions. Hematoxylin counterstaining was used to visualize nuclei. The ATP1A1 expression level in each tissue section was assessed in non-necrotic areas of three separate microscopic fields of view, using 20× magnification. Data represent the mean percentage of ATP1A1+ cells.

## Pharmacological inhibitor treatments

The SFK-specific pharmacological inhibitor PP2 and the Ras inhibitor farnesylthiosalicylic acid (FTS) were purchased from Sigma-Aldrich (St. Louis, MO, USA), dissolved in dimethyl sulfoxide (DMSO), and used at 20 µmol/l and 12.5 µmol/l, respectively, in culture media. These concentrations were implemented based on preliminary analyses of the efficacy of the agents *in vitro*. The cells were treated with FTS or PP2 for 48 h and then harvested for subsequent studies. We used PP2 as a Src inhibitor.

# Supplementary Results

## Knockdown of ATP1A1 expression in GSCs

To investigate the effect of ATP1A1 on GSC growth, we knocked down ATP1A1 expression in GBM GSCs1 and GBM GSCs2 by transfecting them with shRNA targeting ATP1A1. qPCR (Figure S1A) and western blot (Figure S1B) analyses revealed that ATP1A1 expression was markedly decreased in cells transfected with sh-ATP1A1-1 or sh- ATP1A1-2 vector compared with cells transfected with sh-NC vector and non-transfected cells (GBM GSCs1-N and GBM GSCs2-N).

## Re-introduction of ATP1A1 in ATP1A1-knockdown cells restores proliferation and viability

To evaluate the functions of ATP1A1 in GSC proliferation and survival further, ATP1A1-knockdown cells (sh-ATP1A1 cells) were transfected with ATP1A1 cDNA to recover ATP1A1 expression. We then examined GSC proliferation and survival by CCK-8 (Figure S2A), BrdU incorporation assays (Figure S2B), and flow-cytometric analysis (Figure S2C). Cell proliferation and survival were significantly higher in pCMV-ATP1A1 than in sh-ATP1A1 cells. Based on these findings, we concluded that re-transduction of ATP1A1 in the ATP1A1-knockdown cells restored cell proliferation and viability.
